# National Hospital for the Paralysed and Epileptic

**Published:** 1902-06-14

**Authors:** 


### NATIONAL HOSPITAL FOR THE PARALYSED AND EPILEPTIC.

A SPECIAL general meeting of the governors was summoned for May 29th, and at the appointed time, three o'clock, the room was well filled.

The Chairman, Dr. Wace, D.D., in opening the proceedings, remarked that when the present board of management was entrusted with the duty of the administration of the hospital they made it their first obligation to acquaint themselves fully with the condition of all its equipments. This had taken some time, but it had been done with all care and deliberation, and having employed the services of the best advisers possible, they had come to the conclusion that there were certain defects which required remedying at once and which would necessitate an expenditure beyond that authorised. By the ordinary rules the directors were prohibited from laying out more than £1,000 without the special sanction of the governors, and as the proposed improvements would entail an expenditure of over £3,000, the board could not feel safe in undertaking them unless they obtained the authority of the governors. The defects and necessary improvements were of a kind which naturally arose in any great establishment in the course of years, and had to be dealt with at once. Dilapidations would would accrue in all houses in the course of years, and the deficiencies in the hospital were simply of that nature, but they were really serious and such as a surveyor of any private house would consider must at once be remedied.

REPAIRS AND IMPROVEMENTS.

The first matter, that of sanitary work, was, of course, of the greatest importance, and the board felt it was a great advantage that Mr. Langton Cole, who had been architect of the hospital since 1893, should continue to give his services to the new Board of Management. He had observed the condition of the hospital for some time, and on previous occasions had desired improvements to be made, but the time was not then considered to be ripe for them. The state of the drains, as demonstrated in Mr. Langton Cole's report, showed that the defects were not merely ordinary structural defects, but were seriously injurious to the health of the hospital. The outlay required for these absolutely necessary expenses would amount to £820, but in order to keep the drains and pipes in good order in the future, the architect strongly advised a further expenditure of £146 for providing outside ladders, etc.

The matter of fire protection was a comparatively small one. A competent fire engineer had inspected the arrangements of the hospital in this particular, and had made the fire apparatus of the hospital efficient at a cost of £50, and would maintain it in proper working order, and drill the staff and instruct them in the use of the apparatus, at a cost of £10 a year.

The heating system was not so grave a matter as the sanitary work, but still it was an important one. The steam-heating system was thoroughly unsatisfactory throughout. An expenditure of about £900 was recommended as advisable for safety as well as for efficiency and economy.

The electric lighting of the building was not a matter of such vital importance as some of the others, but it was a necessity if the hospital was to be kept up to the highest standard of medical efficiency. It was most important for the purposes of diagnosis, and for the special medical treatment required in the hospital it was most desirable that the electric current should be available at each bed in the wards. The estimate for this work was £1,000.

The board also proposed to expend £100 on a system of telephonic communication between the wards. At present the distance between the wings occasioned much loss of time. The chairman considered it was the duty of the board to do everything that lay in their power in order to assist the resident medical staff in their work. The board thought it was only reasonable that they should leave a margin of 25 per cent. in view of any further expenditure which might prove to be necessary, and they therefore asked for a sum of £4,000, which would, they believed, put the hospital into a position of adequate efficiency. The late board had acquired two adjoining houses, the utilisation of which the present board considered to be quite essential to the completion of the hospital. But the board did not wish to charge the existing funds of the hospital with the cost of these developments, and proposed to make a special appeal to the public for that purpose. The Lord Mayor had kindly consented to allow a meeting to be held in the Mansion House early in July, when the Bishop of London had promised to attend and speak for the hospital, and they hoped they would then be able to raise the money they needed.

But the expenditure for repairs and improvements, they considered, might properly be charged to capital, as it was not required for current expenses, but for permanent improvements. It was of the utmost importance that the improvements should be entered upon with the least possible delay, and the board therefore asked the sanction of the meeting for the sale of funds in order to provide the required sum.

The resolution to this effect having been seconded by one of the governors, Dr. Buzzard rose and, speaking in the name of the medical staff, expressed the gratitude they felt to the board for looking into these matters. There was no possible doubt that these defects required to be dealt with without any further delay; the sanitary work was of vital consequence to the hospital. Fire protection was a most important feature, especially in view of the helpless condition of the patients. As regards the lighting, in the winter time it was almost impossible for those whose duty it was to examine cases to do so with any degree of ease. The hospital was quite behind the age in not having the electric current installed for therapeutic and diagnostic purposes. Dr. Buzzard concluded by again affirming the gratitude of the staff to the board for taking action in the matter.

Mr. Burford Rawlings stated that he was exceedingly glad to hear the Chairman say that the defects and improvements were of a kind which naturally occurred in the course of years. With regard to the finances the speaker ventured to say that he regarded the new departure with great regret. During the whole of the hospital's existence no such proposal had ever been made before as to sell out stock. On every former occasion any such expenditure had been faced and the money obtained. He therefore begged to differ with the wisdom of the proposed step.

The Chairman assured Mr. Burford Rawlings with regard to the financial part of the matter that the board had no intention of regarding their proposed method of raising the necessary funds as a precedent, but it was a method very generally adopted by hospitals. St. George's Hospital proposed to sell out a large sum for current expenses, but in their own case it was simply capital expenditure, which was necessary in order to bring the hospital into a proper state of equipment.

Mr. Danvers Power, the vice-chairman, said that without wishing to criticise the procedure of the old board he would like to point out one or two facts. On January 1st, 1899, there was a surplus of £7,989, which represented the savings of previous years. Now, if there was any virtue in not touching capital, then a surplus must be considered as capital. During the last three years legacies amounting to over £10,000 had been left to the hospital, and these should have been considered as capital and not as investments. This sum was expended by the old board on current expenditure to meet deficits. The speaker found no fault with the old board for this preceeding; they could not have done otherwise. But when the new board came to take up the work, and found that certain repairs and expenditure were necessary, and asked the governors' sanction in order to spend £4,000 out of invested funds, he did not think it was altogether reasonable criticism to say that it would be far better if they respected the sanctity of invested funds. These funds, the new board found, had not been invested very strictly.

Mr. Burford Rawlings, in replying to Mr. Danvers Power, stated that the old board worked on an intelligible principle and invested every legacy, and on no single occasion was any of that money touched until the rebuilding of the hospital began, when the board considered it advisable that the funding of legacies should cease, and that they should be used for the extension of the freehold. With regard to the investing the surplus of one year, any gentleman who was acquainted with the finances of hospitals would know that that was an impossibility, the incomes of hospitals being so fluctuating. During the last three years the late board had laboured under great difficulties which had rendered it impossible for them to appeal to the public for money in the usual way.

The resolution empowering the Board of Governors to sell funds for the proposed expenditure was then passed with one dissentient vote.

